# Shaping ultrasound in midwifery: towards an evidence-based training framework for enhanced prenatal care

**DOI:** 10.1007/s00404-024-07558-3

**Published:** 2024-05-21

**Authors:** Julia Groos, Adeline Walter, Agnes Wittek, Brigitte Strizek, Ulrich Gembruch, Florian Recker

**Affiliations:** Department of Obstetrics and Prenatal Medicine, University Hospital Bonn, Venusberg Campus 1, 53127 Bonn, Germany

**Keywords:** Midwifery, Ultrasound education, Obstetrical imaging, Training

## Abstract

**Background:**

Academic advancement of the midwifery profession highlights the need to establish standardized qualifications in obstetric ultrasound diagnosis, being a central part of prenatal care. Thus, introduction of an evidence-based training program is warranted. We aimed to reviewed curriculum designs used in midwifery ultrasound education.

**Methods:**

A systematic literature research was conducted. Embase, PubMed and Google Scholar database was reviewed for publications using the terms “[midwife], [midwives], [midwifery students], [obstetric ultrasound], [midwife sonographer] and [education], [teaching], [program], [course], [curriculum] and [learning]”. Papers with full description of curriculum designs or educational programs on obstetrical ultrasound for midwives were included and scrutinized against pre-defined criteria according to the PICO (Population, Intervention, Comparator, Outcomes) scheme.

**Results:**

29 publications were included. Studies demonstrated a significant disparity according to course concepts being used. Differing parameters included: Duration, structure, learning approaches, course content, examination concepts and target groups (practising midwives vs. midwives in education).

**Conclusion:**

An evidence-based ultrasound educational program for midwives remains to be developed, including further educational guidelines. Clinical applications by midwives, as well as the distinctions from medical practise, particularly in terms of legal considerations, needs to be defined.

## What does this study add to the clinical work

**Table:** 

Recognizing the growing importance of ultrasound in prenatal care, implementation of this diagnostic tool into midwifery profession is essential. This review proposes the development of an evidence-based ultrasound educational program. Disparities in current training programs are demonstrated by underlining the lack of a standardized curriculum. Leading to a high variability in the proficiency and confidence among midwives performing ultrasound examinations, a standardized training program is warranted.	

## Introduction

Increased sophistication in ultrasound technologies has enabled ultrasonographic diagnostics becoming an essential part in pregnancy care. From the initial focus on screening programs and evaluation of fetal and placental assessment, published studies had further demonstrated the feasibility and utility of ultrasound diagnostics during the intra- and postpartum course [1–6].

Given the continuous increase in out-of-hospital births, which are amongst others home births, or births in midwife-led institutions corresponding to independent births centres, the utilization of ante- and intrapartum ultrasound technology by midwives has the potential to decrease intra- and postpartum complications [7, 8]. In consequence, ability by midwives to promptly identify and evaluate potential risks, facilitates a timely hospital transfer, thus enhancing maternal and neonatal outcomes.

The regular incorporation of ultrasound into midwifery practise becomes achievable by the growing availability of point-of-care ultrasound devices. The use of small and handy devices, possess midwives the flexibility to perform examinations in direct patient contact and regardless of where they are needed [9–11].

Within the context of the ongoing academization of the midwifery profession, one of the primary objectives is the qualitative and competence-enhancing further development of midwifery. This includes adapting to new opportunities in diagnostics and preventive measures, made possible by the medical progress [9–11]. Hence, innovative practices, such as ultrasound, can enhance the midwife's prescribed activities, presenting valuable opportunities to progress towards a comprehensive ante-, intra- and postpartum obstetric care.

However, as the level of expertise mainly determines for an adequate reproducibility and for a correct interpretation of data, evidence-based training programs are needed. We aimed to present the current state of obstetric ultrasound education for midwives in the context of introducing an evidence-based German training program. For this, we performed a systematic literature review and compared previous published data, regarding different didactic methods, course contents (incl. ante-, intra- and postpartum ultrasound), learning approaches, examination concepts, target groups (practising midwives vs. midwives in education), course durations and course formats. Current developments and challenges arising by the ongoing process of academization and the draft amendment of the Midwifery Act were further discussed.

## Methods

### Search strategy

**Fig. 1:**
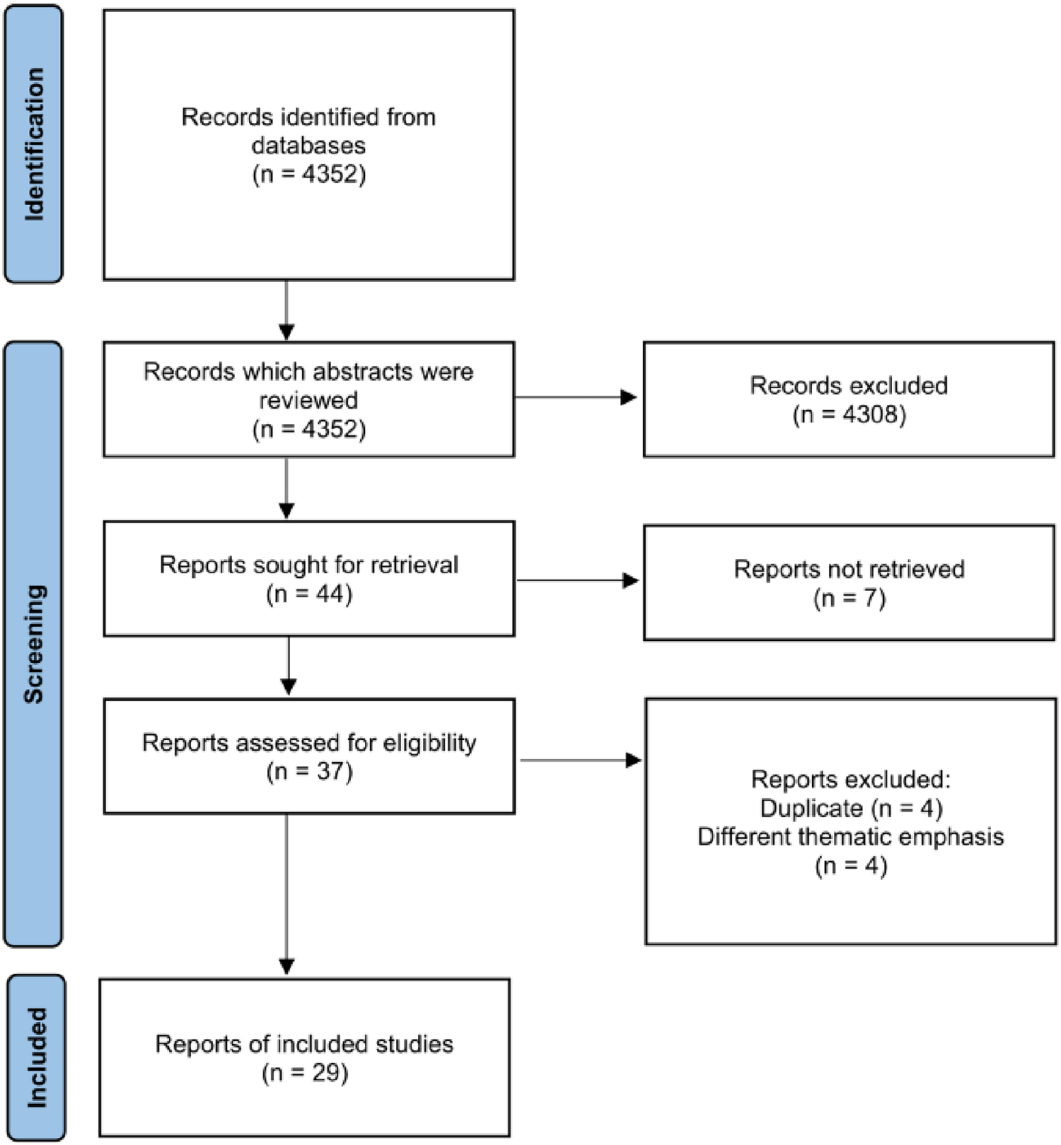
Study selection process according to Preferred Reporting Items for Systematic Reviews and Meta-Analyses (PRISMA) guidelines

In the process of conducting the systematic literature search, the updated Preferred Reporting Items for Systematic Reviews and Meta-Analyses (PRISMA) statement for reporting systematic reviews and meta-analyses of studies was followed (Fig. 1) [12, 13].

In April 2023, the PubMed, Embase and Google Scholar databases were searched for relevant publications in English, French or German on the topic of midwifery education in obstetric ultrasound using the keywords [midwife], [midwives], [midwifery students], [obstetric ultrasound], [midwife sonographer] and [education], [teaching], [program], [course], [curriculum] and [learning]. To formulate a specific search query, keywords were partially linked with operators as follows: “obstetric ultrasound”, “midwifery education programs”, “midwife sonographer”, “midwifery students”, allintitle: midwives obstetric ultrasound. Titles and abstracts were screened by three authors in an unblinded manner for compliance with inclusion criteria. Hence, relevant study characteristics had been specified in advance using the PICO (Participants, Intervention, Comparator, Outcomes) process (Table 1) [12]. A comprehensive systematic review was conducted on the corpus of literature pertaining to the instruction of midwives in the domain of obstetric ultrasound. The inclusion criteria were broad, encompassing retrospective and prospective research studies, surveys, clinical guidelines, professional recommendations, and other scholarly works that addressed aspects of midwife education in obstetric ultrasound. Specifically, the review focused on literature detailing the educational levels of midwives, their learning objectives, curriculum design and structure, pedagogical strategies, instructional materials, methods of assessment, challenges encountered in previous implementations, potential opportunities for advancement, and solutions, as well as the incorporation and utility of specialized ultrasound technology and instrumentation, where applicable. The selection process did not discriminate based on the disciplinary backgrounds of the authors. Exclusions were made for articles not written in German, French, or English, duplicates, those divergent in theme, and any without accessible full-text versions. For the purpose of data extraction, three researchers (J.G., F.R., and A.W.) conducted a thorough double review of the full texts of all identified sources. The article is structured around key questions, which served as a framework for guiding the systematic extraction and synthesis of data.

**Table 1:** Inclusion characteristics and target aspects

Participants	Student midwives, practising midwives, (in)experienced in midwifery, (in)experienced in ultrasound
Intervention	Obstetric ultrasound training
Comparator	Different approaches and ideas
Outcomes	Target groups, contents, course formats, teaching methods, examination modalities and barriers in obstetric ultrasound training for midwives
Study design	Original study or reports on prospective and retrospective studies, interventional studies, observational studies and cross-sectional studies

### Registration

This systematic literature review has been successfully registered to the international prospective register of systematic reviews PROSPERO on 10/7/23 with the registration number: CRD42023440461.

## Results

**Fig. 2:**
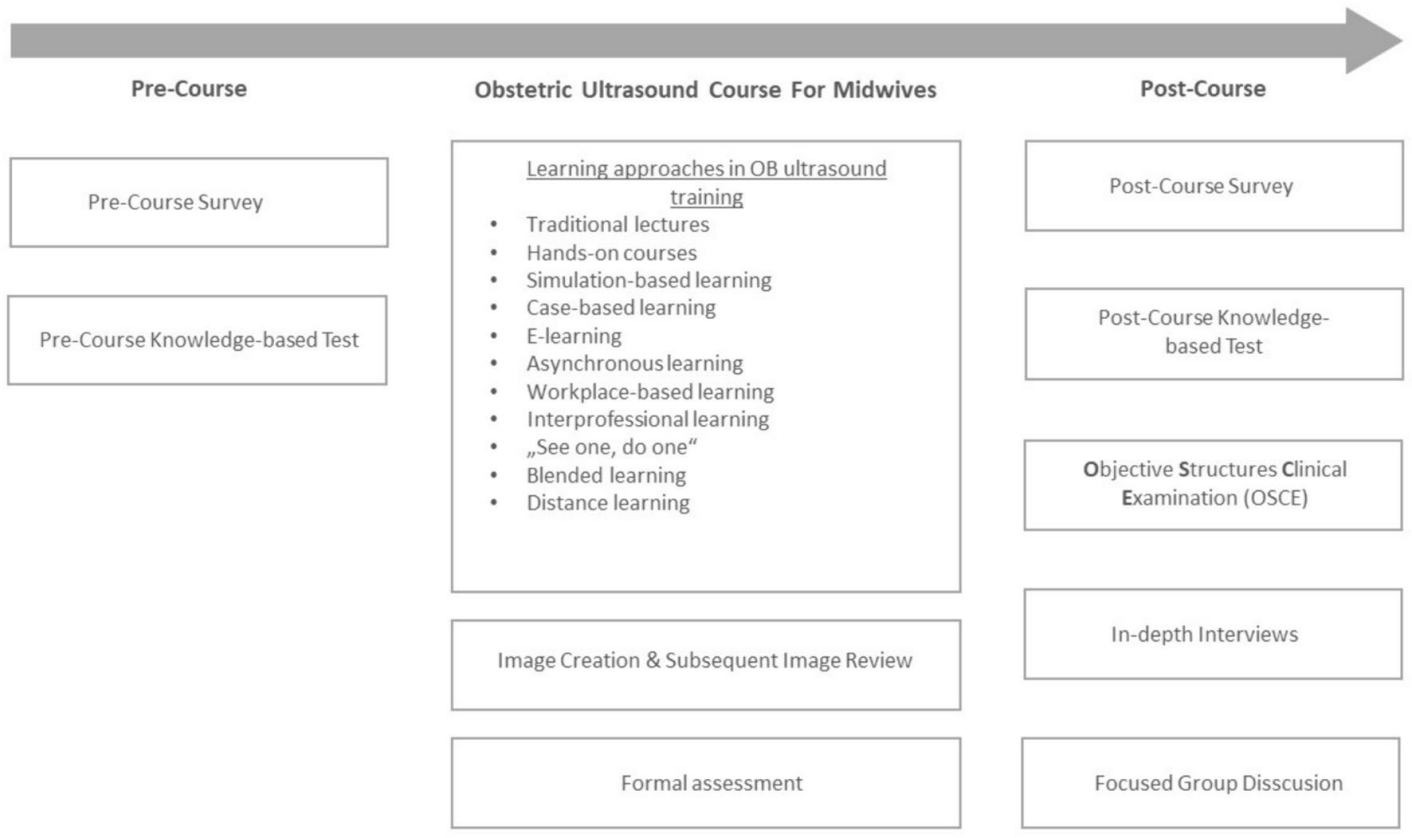
Summary of different learning approaches and assessment formats in obstetric ultrasound training for midwives

Finally, 29 articles, comprising 4 reports and 25 studies, were acceptable for consideration in the literature selection (see Fig. 1). Tables 2, 3 and 4 list all reports and studies with respect to their PICO criteria and Fig. 2 illustrates the geographical parts of the world from which the included publications originate.

**Table 2:** Included studies and their characteristics

Author(s)	Title	Participants	Intervention	Comparison	Outcomes	Study design	Year of publication
Bentley et al. [16]	Evaluation of an Obstetric Ultrasound Curriculum for Midwives in Liberia	Midwives (*n* = 31)	• 1 week ultrasound curriculum – Didactic components – Practical components – Supervised patient encounters • Pre -, post-course knowledge test • Pre-, post-course survey • OSCE	• Pre-, post-course knowledge assessment • Pre-, post-course survey • Pre-, post written & practical assessment after 1 year	A 1 week curriculum led to increased short- and long-term knowledge and comfort	Prospective study	2015
Shaw-Battista et al. [28]	Interprofessional obstetric ultrasound education: Successful development of online learning modules; Case-based seminars; Skills labs for registered and advanced practise nurses, midwives, physicians, and trainees	Certified nurse-midwives (CNMs), nurses, other advanced practise nurses, physician assistants, family practise physicians (*n* = 72)	• Interprofessional obstetric ultrasound course – Asynchronous online modules – Case-based seminar – Skills lab • Pre-, post-course knowledge test	• Pre-, post-course knowledge test	Interprofessional obstetric ultrasound education can enhance trainees’ understanding of collaborative practise and clinical imaging skills	Prospective study	2015
Viner et al. [14]	Training in Ultrasound to Determine Gestational Age (TUDA): Evaluation of a novel education package to teach ultrasound-naive midwives basic obstetric ultrasound in Malawi	Midwives (*n* = 29)	• 10 day ultrasound program – Didactic components – Hands-on practise (Supervised Practice, Formal Assessment) – Simulation session – Small group session • Remote supervision and image review over 3 M • Pre-, post-course test • Pre-, post-course questionnaire • OSCE	• Pre-, post-course test • Pre-, post-course questionnaire • Image review by experienced obstetric sonographers • Pre-, post-course written and practical assessment after 3 M	The TUDA training program proves to be an efficient approach to equip midwives with fundamental skills in obstetric ultrasound within a 2 week timeframe	Mixed methods quasi-experimental trial	2022
Shah et al. [22]	Efficacy of an ultrasound training program for nurse midwives to assess high-risk conditions at labor triage in rural Uganda	Nurse/nurse midwives (*n* = 23)	• Point-of-care ultrasound training program – Didactic components in small groups – Hands-on practise – Supervised mock enrolment • Pre, -post-course survey • OSCE • Image review • Qualitative interview	• Pre, -post-course survey • Image review by ultrasound experts	A 2 week intensive training, featuring short lectures and extended hands-on practise, followed by ongoing local mentoring, enhances confidence and POCUS skills for identifying critical labor conditions	Mixed-methods study	2020
Viner et al. [25]	Midwife-led ultrasound scanning to date pregnancy in Malawi: Development of a novel training program	Midwives (*n* = 29)	• Ultrasound training program – Lectures – Supervised hands-on practise – Small group session – Simulation – Formal assessment • OSCE	• Measurements obtained by trainer	Basic perinatal ultrasound fundamentals can be effectively taught within a relatively brief timeframe	Prospective study	2022
Mubuuke, A.G., Nassanga, R. [24]	Point of care obstetric ultrasound knowledge retention amongst mid-wives following a training program: a prospective cohort pilot study	Midwives (*n* = 11)	• 6 week POCUS curriculum – Didactic components – Hands-on practise • Longitudinal supervision and training over 6 M • Pre-, post-course self-rated knowledge tool • Post-course exam • Post-course questionnaire	• Pre-, post-course self-rated knowledge tool • Post-course exam (immediate, 6 M) • Post-course questionnaire (immediate, 6 M)	Point of care obstetric ultrasound training for midwives can lead to satisfactory levels of knowledge retention	Prospective cohort pilot study	2023
Bidner et al. [20]	Evaluation of antenatal point-of-care ultrasound training workshops for rural/remote healthcare clinicians: a prospective single cohort study	Midwives (*n* = 25), general practitioners (*n* = 16)	• Pre-workshop survey • 2 day antenatal ultrasound workshop – Lectures – Practical session – Simulation • Pre-, post-course knowledge assessment • 3- and 6 month post-workshop survey • Post-workshop evaluation • Online group mentoring session • OSCE • 2 day follow-up training workshop after 12 M	• Pre- and post-course knowledge assessment • 3- and 6 month post-workshop survey	Intensive 2 day workshops can furnish clinicians with valuable prenatal POCUS skills	Prospective study	2022
Hall et al. [17]	Analysis of an obstetrics point-of-care ultrasound training program for healthcare practitioners in Zanzibar, Tanzania	Midwife/nurse (*n* = 8), clinical officer (*n* = 3), medical officer/physician (*n* = 2)	• 2 week antenatal ultrasound course – Lectures – Supervised hands-on practise – Ultrasound case review – Workplace-based learning over 6 months • Pre-, post-course exam • Mid-, post-course OSCE	• Pre-, post-course written test/exam • Mid-, post-course OSCE	Despite a relatively low completion rate, trainees demonstrated significant improvements across all measures following the training program	Prospective study	2021
Nathan et al. [31]	Screening Obstetric Ultrasound Training for a Five-Country Cluster Randomized Controlled Trial Problem	Ultrasound-naïve, nonphysician healthcare professionals	• 10 day course – Didactic components – Supervised and independent hands-on practise – Case studies – Extensive quality assurance activities – Educational outreach visits – Workplace-based learning and exam review over 3 months – Remedial training	• Remote examination review	-	Cluster-randomized controlled trial	2014
Kozuki et al. [33]	Accuracy of home-based ultrasonographic diagnosis of obstetric risk factors by primary-level health workers in rural Nepal	Auxillary nurse/midwives (*n* = 3)	• Two one week ultrasound training – Lectures – Practice – Demonstration – Home visits • Image review	• Home visit scans by second auxillary nurse midwives • Image review by two sets of reviewers	Primary healthcare providers can perform accurate third-trimester obstetric ultrasound examinations to identify obstetric risk factors effectively, even with minimal training	Prospective study	2016
Baj et al. [30]	Obstetric ultrasound education for the developing world: A learning partnership with the World Federation for Ultrasound in Medicine and Biology (WFUMB)	Midwives and other health workers	• Ultrasound program with blending learning methods – 10 e-learning sessions – Hands-on training • Evaluation survey • Feedback	-	Besides satisfaction with the e-learning program, there are some difficulties that affect the learning experience	Prospective pilot study	2015
Kimberly et al. [40]	Focused maternal ultrasound by midwives in rural Zambia	Midwives (*n* = 21)	• Ultrasound course • Didactic components • Hands-on components • Supervised scanning sessions • OSCEs • Image and datasheet review • Follow-up questionnaire	OSCE at 2 and 6 months Scan review by ultra- sound fellowship trained physicians	Midwives in rural Zambia can retain basic obstetric ultrasound skills over six months and continue using ultrasound in a one-year assessment	Prospective study	2010
Kim et al. [23]	An evaluation of obstetric ultrasound education program in Nepal using the RE-AIM framework	Non-physician (e.g. nurses, midwives, paramedical staff), Physicians (OB-GYNs, pediatricians, radiologists, medical doctors from different specialties) (*n* = 228)	• 2 day intensive ultrasound education program – Lectures – Case discussion – Experience sharing – Live demonstration – Program evaluation • Administrative record • Pre-, post-course test • Pre-post-course survey • Online follow-up survey • In-depth interview	• Pre-, post-course test • Pre-, post-course survey • Online follow-up survey	Participant’s knowledge and self-confidence in ultrasound techniques were improved and there was a great potential for adoption and maintenance	Mixed-method study	2021
Nathan et al. [29]	Evaluation of focused obstetric ultrasound examinations by health care personnel in the Democratic Republic of Congo, Guatemala, Kenya, Pakistan, and Zambia	Healthcare professionals. (midwives, nurses, radiographers, and medical officers) (*n* = 41)	• 2 week course in basic obstetric ultrasound – Didact session – Supervised hands-on training – 12 weeks oversight pilot phase – Remedial training • Examination review and feedback • Scanning skills test • Written test	• Examination review by either University of Washington’s Department of Radiology (UW) or by local reviewers	Trainees with no prior ultrasound experience achieved a high concordance in ultrasound diagnoses with reviewers when working independently	Cluster-randomized trial	2017
Johnston et al. [19]	Insights into an innovative point-of-care ultrasound curriculum for Ontario primary maternity care providers	Midwives (*n* = 17)	• Initial modules, formative quizzes • POCUS education course – Online modules – Hands-on workshop – Simulation – Supervised clinical practicum guided by a logbook • OSCE • Evaluation survey	• Logbook entry evaluated by a sonography preceptor	The midwives mentioned that the course provided them with a thorough understanding of the knowledge and clinical skills needed to use POCUS in antenatal care	Case study	2022

**Table 3:** Included studies focused on simulation and further technological aids

Author	Title	Participants	Intervention	Comparison	Outcomes	Study design	Year of publication
Zimmermann et al. [32]	E-learning et simulation en échographie focalisée pour la formation continue des sages-femmes en salle de naissance	Midwives (*n* = 33)	• Ultrasound program with blending learning methods – E-learning platform – 1 day simulation-based hands-on workshop • Pre-, post-simulation practical assessment • Pre-, post-course knowledge test (T1-T3) • Satisfaction questionnaire	• Pre-, post-simulation practical assessment • Pre-, post-course knowledge test (T1-T3)	Combining e-learning and practical simulation workshops in obstetric ultrasound training is a beneficial and acceptable approach for practising midwives	Descriptive cohort study	2019
Vinayak et al. [18]	Training midwives to perform basic obstetric Point-of-Care Ultrasound in rural areas using a tablet platform and mobile phone transmission technology—A WFUMB COE project	Midwives (*n* = 3)	• Ultrasound curriculum with blending learning methods and mobile learning – E-learning module with subsequent test – Lectures – Hands-on practise – Provisional report – “See one, do one” • Exit examination (written, practical) • Questioning • Image and report review via teleradiology system	• Final outcome of pregnancies • Pre-, post overall turnaround time • Image and report review by radiologists	Training midwives in POCUS to use ultrasound tablet devices and transmit images and reports via the internet to radiologists is valuable for accuracy	Prospective cross-sectional study	2017
Jemal et al. [27]	Implementation and evaluation of a pilot antenatal ultrasound imaging programme using tele-ultrasound in Ethiopia	Midwives (*n* = 7), Clinical Health officers (*n* = 5), Nurses (*n* = 2)	• 3 week ultrasound curriculum using a tele-ultrasound platform – Didactic component – Supervised practical component – Real-time support and feedback via tele-ultrasound platform • Pre-, post-didactics test • Practical assessment • Focus group discussion • In-depth interview • Post-course questionnaire for participants and patients • Interview	• Image interpretation of obstetricians • Pre-, post-didactics test	Succeeded in training healthcare providers to conduct antenatal ultrasound examinations and provided support using a tele-ultrasound platform	Mixed-methods study	2022
Gueneuc et al. [21]	Impact of sonography simulation in the training of midwifery students	Student midwives (*n* = 40)	• Ultrasound course in 2 groups 1) no simulator training prior to practise training 2) simulator training prior to practise training – Ultrasound internship – Ultrasound training on simulator • Evaluations (E1-E3) on simulator: OSAUS, Image assessment	• Group 1 & 2 – Image assessments according quality criteria – Objective Structured Assessment of Ultrasound Skills (OSAUS)	The addition of simulator training to clinical ultrasound instruction seems to have a notable impact on enhancing obstetrical ultrasound proficiency	Prospective study	2019
Chan et al. [38]	The use of three-dimensional ultrasound does not improve training in fetal biometric measurements	Midwives (*n* = 10)	Ultrasound course in two groups 1) 2D in ultrasound fetal biometry assessment 2) 3D in ultrasound fetal biometry assessment – Briefing session – 4 supervised hands-on sessions – 2 practical evaluation sessions	Group 1 & 2 – Set of measurements by the trainer – Time required for completion – Image quality review	An obvious advantage of 3D technology compared to traditional 2D imaging in basic biometric measurement training could not be demonstrated	Prospective study	2011
Le Lous et al. [36]	Improving students’ ability to perform a standardized foetal biometry plane using ultrasound simulators	Midwives (*n* = 9), specialist residents (*n* = 21)	• 1-h ultrasound course with simulators • Pre-, post-training practical assessment	• Pre-, post-training practical assessment	Obstetrical ultrasound simulation offers an effective way to improve skills and expedite learning, even with brief exposure and without the need for volunteers	Prospective single-centre study	2017
Solano et al. [35]	Asynchronous telemedicine with ultrasound: Improving maternal health in developing countries	Midwife (*n* = 1), OB/GYN specialists (*n* = 3)	• Deployment of a web-based asynchronous telemedicine system – 4 h didactic instruction – 3 × 15-min orientation sessions • Pre-, post-deployment test • Satisfaction survey	• Pre-, post-deployment test	It is feasible to expand routine prenatal ultrasound access to underserved populations and to coach a midwife to achieve obstetric ultrasound skills through a web-based educational system	Prospective study	2009
Anderson et al. [39]	The midwife’s assistant: Designing integrated learning tools to scaffold ultrasound practise	Midwives (*n* = 10), nursing and midwifery educators (*n* = 2), ultrasound instructors (*n* = 5), traditional birth attendant (*n* = 1), community health worker (*n* = 1), rural mothers (*n* = 59)	• Fieldwork trips – Observation of novice midwife sonographers – Focus groups – Individual interviews – Usability tests of the system	-	In addition to having access to reference materials during exams, midwives require in-depth learning materials that can be accessed outside of a medical examination setting	Prospective study	2012
Grandjean et al. [34]	Fetal biometry in ultrasound: A new approach to assess the long-term impact of simulation on learning patterns	Midwifery students (*n* = 25), residents (*n* = 36)	• Ultrasound workshop in 2 groups 1) Simulation group (before, early, late) 2) Control group – 2 h theoretical presentation – 3 h supervised hands-on workshop • Pre-, post-course practise assessment (OSAUS) • METHOD Logbook platform	• Different subgroups – Pre-, post-course practise assessment (OSAUS) – Set of measurements of the supervisor – Review on METHOD Logbook platform	The study did not show long-term benefits from SBE training, however, SBE showed a positive impact on raising the minimum skill level when included early in a practical course	Prospective study	2021
Osborne et al. [37]	The effectiveness of simulation training in the teaching of skills required for sonographic fetal assessment in mid-trimester pregnancy to novices: A pilot study	Practising midwives (*n* = 9), resident medical officer (*n* = 1)	• 5 week training simulation ultrasound programme – Pre-training reading – 3 simulator practical training and testing sessions – Practical live model testing session • Pre-, post-course practical assessment • Image and measurement review	• Pre-, post-course practical assessment • Live model testing • Reference measurements by accredited sonographers	There were no significant differences in participant performance between simulator and live model tests, suggesting that simulator skills can predict real-patient performance, with transferable skills to more realistic settings	Prospective observational study	2016

**Table 4:** Included reports

Author(s)	Title	Type of publication	Year of publication
Robinson, C. [26]	ASUM outreach CAHPU midwives training course	Article	2017
Johnston, BK [15]	Evaluation of point-of-care ultrasound training for midwives	MSc. Thesis	2021
Uys, N. [41]	A health systems engineering approach to meeting the demand for skilled foetal ultrasound services in the Boland/Overberg public health district	Final year project	2010
DeStigter, K. [42]	mHealth and developing countries: A Successful Obstetric Care Model in Uganda	Research article	2012

### In which stage of education midwives are trained in obstetric ultrasound?

The pedagogical orientation of obstetric ultrasound training programs for midwives is predominantly focused on practitioners with foundational competencies in midwifery, yet devoid of prior ultrasound experience [14, 15]. It is observed that a significant proportion of midwives engaging in obstetric ultrasound education exhibit minimal baseline proficiency in ultrasonography, often with negligible formal training and lacking routine application in their professional practise [16, 17]. In terms of clinical experience, a substantial body of research includes midwives with extensive practise in the field of midwifery. For instance, Viner et al. have documented the involvement of midwives possessing credentials in nursing and midwifery, with an average midwifery experience of 10.5 years, albeit without prior exposure to ultrasound technology [14]. Conversely, certain educational programs also cater to midwives with considerably less professional experience, such as under five or three years [15, 18].

For midwives, participation in continuing education courses on ultrasound diagnostics represents a significant enhancement of their ultrasonographic skills and knowledge, augmenting their existing clinical competencies [19]. Throughout the duration of these training courses, midwives are either relieved from their clinical responsibilities or they undertake the training concurrently with their standard duties [17, 20].

Distinct from the prevalent focus of research on training practising midwives in obstetric ultrasound, Gueneuc et al. have highlighted the enrollment of student midwives, specifically those in the concluding stages of their midwifery education, in ultrasound training programs [21].

### What intention leads to develop and implement the obstetric ultrasound courses for midwives?

The initiative to develop and implement ultrasound training programs for midwives is predominantly observed in low- and middle-income countries (LMICs) (see Fig. 2), where access to ultrasound services for pregnant women is often limited [14]. Geographic challenges, including extensive distances and inadequate transportation infrastructure, compound the difficulty of accessing obstetric care, imposing economic burdens [17]. These regions are further characterized by a significant dearth of trained personnel and insufficient resources for hospitalization, alongside deficiencies in antenatal care and elevated rates of maternal and fetal mortality [17, 20]. In LMICs, congenital anomalies account for approximately 5–7% of perinatal mortality rates, suggesting that improved access to obstetric ultrasound could enhance the detection of high-risk conditions and improve maternal and neonatal outcomes [22, 23].

The strategic training of midwives in obstetric ultrasound seeks to mitigate the impact of sonographer shortages. Midwives, given their direct involvement in the care of pregnant women, emerge as the ideal candidates for the acquisition and application of obstetric ultrasound skills, with the primary goals of augmenting clinical care, expediting the management of expectant mothers, and reducing incidences of preventable deaths [22, 24–26].

Improving healthcare and ultrasound services in areas with limited resources, WHO recommendations suggest to let trained non-physician providers, to handle specific physician tasks. With antenatal ultrasounds in these areas being performed by midwives, further WHO recommendations like at least one ultrasound scan before 24 weeks of pregnancy might be fulfilled [25, 27].

In addition, ultrasonography serves as a more accurate alternative to traditional methods of gestational age estimation, such as abdominal palpation and calculation based on the last menstrual period [25]. This non-invasive, cost-effective approach not only enhances the accuracy of physical assessments and clinical decision-making but also facilitates timely referrals to specialized care centers [20, 23, 24, 28].

The introduction of point-of-care ultrasound (POCUS) training programmes addresses the specific needs of patients in rural settings through the use of portable equipment and the capability for bedside examinations. This strategy enables prompt and appropriate prenatal care planning and referrals for urgent cases [20].

### Which learning objectives should be achieved at the end of the obstetric ultrasound course?

Within the scholarly literature, there exists a consensus regarding the objectives of basic obstetric ultrasound training for midwives. Such courses are designed to acquaint midwives with the fundamentals of ultrasound technology, including the operational setup of ultrasound machines, maneuvering of the equipment, and generation of high-quality diagnostic images. The curriculum extends to foundational knowledge in ultrasound physics, and an overview of the technology’s capabilities and limitations [16, 25, 28].

A critical component of the training involves fostering effective communication skills and professional conduct during patient interactions, emphasizing the significance of the midwife's role in prenatal ultrasound examination. This aspect of training underscores the importance of patient-centered care and safety [24, 25].

The educational objectives also encompass the acquisition of skills necessary for conducting various obstetric ultrasound examinations, with a particular focus on fetal biometry for gestational age estimation. Key biometric indicators include the biparietal diameter (BPD), head circumference (HC), abdominal circumference (AC), crown-rump length (CRL), and femur length (FL) [16, 25, 29]. In addition, midwives are educated on identifying fetal presentation, vitality, and number, as well as placental positioning and amniotic fluid volume [24].

Participants are trained to perform comprehensive assessments of maternal and fetal anatomy across all trimesters, enabling the identification of both normal and pathological conditions [30]. This capability is vital for the early detection of high-risk pregnancies and conditions such as placenta previa, oligohydramnios, or malpresentation, facilitating timely referral to specialized care [29, 30].

Furthermore, the curriculum may include elements of interprofessional collaboration, aiming to enhance the participants’ comprehension of cooperative healthcare delivery, task delegation, and shared responsibilities. This aspect of training is intended to foster a multidisciplinary approach to patient care, improving outcomes through integrated team efforts [28].

### Which educational approaches are currently being explored?

**Fig. 3:**
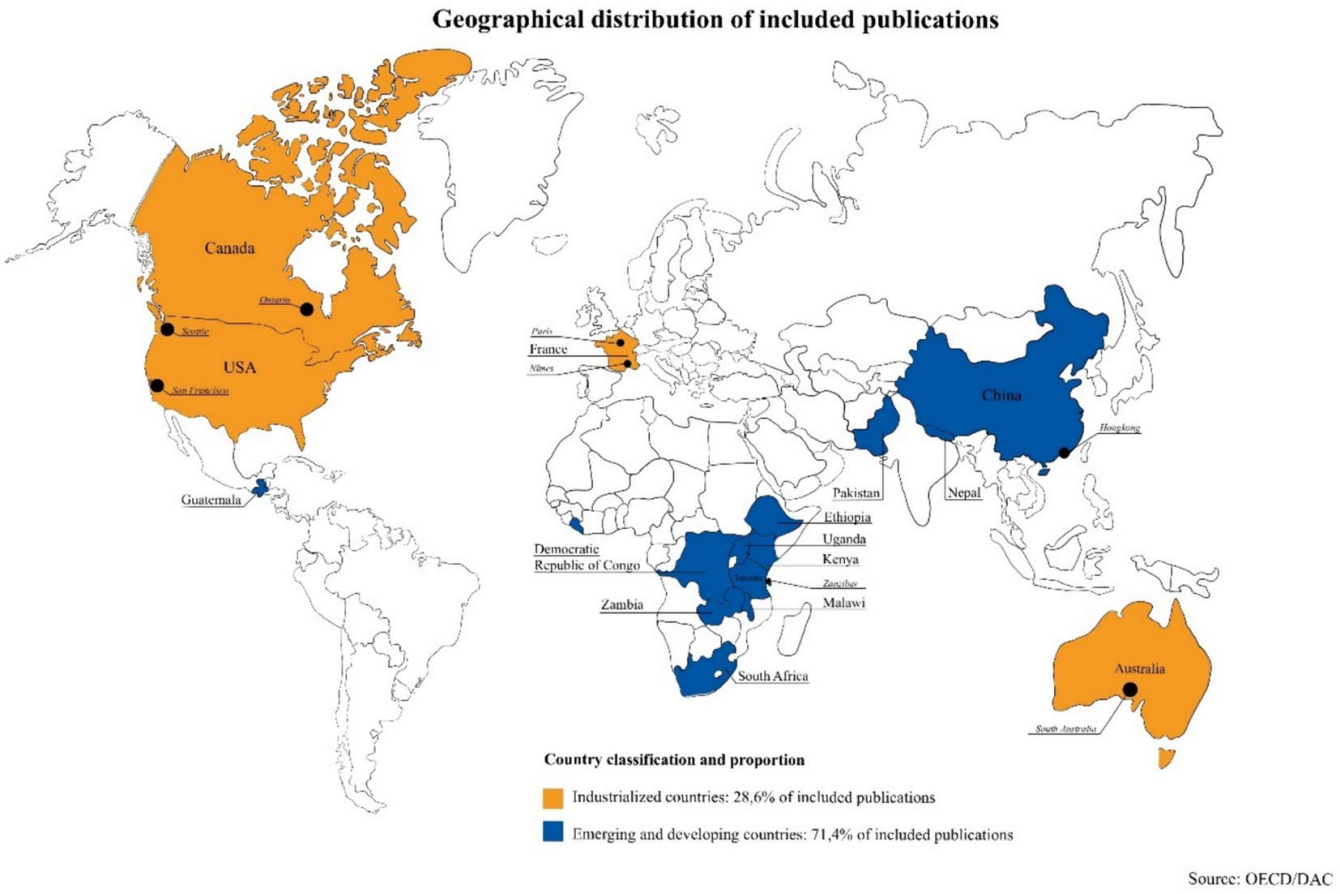
Geographical distribution of included publications

While numerous studies exhibit congruent objectives in the instruction of basic obstetric ultrasound skills to midwives, diverse pedagogical strategies are discernible across the literature (see Fig. 3). A common framework involves a bifurcated approach to teaching, intertwining theoretical knowledge with practical application. Despite variations in the emphasis and allocation of these components across different programs, a predominant focus on hands-on experience is noted. [17, 20].

The instructional trajectory typically initiates with didactic sessions, transitioning through a spectrum of educational methodologies that include lectures, case discussions, and live demonstrations [18, 23]. The incorporation of case-based seminars facilitates the application of theoretical knowledge to real-world scenarios, fostering critical thinking and dialogue [28, 31]. Course formats vary, with some programs delivering theoretical content in-person, while others explore the efficacy of digital platforms or self-guided study through electronic modules [18, 22, 24].

Zimmermann et al. highlighted the use of the “Esimgo” e-learning platform, which organizes content into sequentially structured modules, enhancing the practical training phase [32]. Similarly, Shaw-Battista et al. utilized asynchronous online modules to accommodate participants from remote locations, underscoring the adaptability of digital learning environments [28].

Practical training, often constituting the majority of the course duration, includes direct interaction with ultrasound equipment and live scans on pregnant volunteers, occasionally supplemented by simulation-based experiences. This phase emphasizes the importance of mentorship, with trainers providing real-time feedback and support [14, 28, 31, 32]:

Following practical instruction, a clinical phase enables the application of acquired skills in a professional setting, with ongoing expert supervision and feedback serving as a mechanism for continuous learning and quality assurance. This period, typically ranging from 3 to 6 months, represents a crucial phase of workplace-based learning [14, 17, 18].

Blended learning approaches, combining digital modules with in-person instruction, offer a flexible foundation for theoretical understanding, subsequently reinforced through practical and clinical experiences [18]. Innovative formats, such as live remote guidance during ultrasound sessions and interprofessional courses, highlight the evolving nature of ultrasound education for midwives [27, 28].

The duration of these courses varies significantly, from intensive 2 day workshops to multi-week programs, reflecting the diversity in educational design and objectives [23, 24, 26]. Support mechanisms, including online forums and diagnostic flowcharts, provide additional resources, facilitating a comprehensive learning experience and ensuring the effective application of ultrasound skills in clinical practise [20, 31].

### Which course formats and components fit the best for educating midwives in obstetric ultrasound?

The majority of research focuses on single-instance obstetric ultrasound training programs, spanning from a few days to several weeks, incorporating both theoretical knowledge and practical skills development [16, 24]. Various studies, including those by Bentley et al. with a 1 week course, Viner et al. with a 10 day course, and Kim et al. with a 2 day course, demonstrate the effectiveness of these formats in enhancing midwives’ knowledge, confidence, and clinical proficiency in ultrasound application. Results from these investigations indicate significant gains across both extensive and concise training durations [14, 16, 23].

However, certain analyses reveal limitations regarding the adequacy of training duration for skill competency. Viner et al. suggested that a 5 day course fell short of fostering sufficient competency, recommending a minimum of 10 days to allow for ample hands-on experience [25]. This perspective is echoed by Shaw-Battista et al., who advocated for increased scanning practise through additional sessions or extended training lengths to bolster educational outcomes and skill retention amongst midwives [28]. Moreover, the implementation of refresher courses is identified as a beneficial strategy for reinforcing knowledge [20].

Johnston et al. underscored the critical role of integrating both theoretical and practical elements in the curriculum for teaching point-of-care ultrasound (POCUS) to midwives [15]. Hands-on training is emphasized to be a very valued component of ultrasound courses [28]. Programs rich in practical training are noted for their efficacy in translating complex content into clinically relevant skills [20]. Furthermore, the frequency of practise, as evidenced by the number of scans performed during training, correlates positively with improved knowledge acquisition and self-efficacy [24].

The integration of simulation-based learning alongside clinical training is gaining prominence, particularly in obstetrics and fetal medicine. This blend of simulation and clinical practicum is shown to markedly enhance learner outcomes and competency in performing obstetric ultrasound independently on patients [21].

Inclusion of online modules within the training framework has received favorable evaluations, with participants particularly valuing the availability of preparatory materials, especially beneficial for those facing geographical constraints [20, 28].

Additional components contributing to skill enhancement include structured in-course supervision to provide essential support and feedback, and the involvement of experienced sonographers during practical sessions, offering invaluable expertise to midwife trainees [15, 24, 28].

### How are potential increases in knowledge and newly acquired skills measurable and assessable at the end of the course?

To evaluate the effectiveness of obstetric ultrasound training programs, a variety of assessment methodologies are employed, including multiple choice tests, questionnaires, and objective structured clinical examinations (OSCEs). It is common for research to integrate multiple forms of evaluation, facilitating a comparative analysis of participant’s knowledge and skills before and after the course [14, 16] (see Fig. 3).

Initial knowledge levels are often gauged via pre-course assessments, typically comprising multiple-choice questions that cover essential domains such as the theoretical underpinnings of ultrasound technology, obstetric anatomical understanding, and patient communication strategies. Pre-course surveys may also be conducted to ascertain participants’ baseline comfort with and experience in ultrasound, alongside their expectations regarding the training’s impact. Post-course evaluations, mirroring the pre-course assessments or incorporating specific content-related queries, enable a detailed appraisal of theoretical learning outcomes [16, 17, 20].

Practical proficiency is predominantly assessed through OSCEs, conducted after the course's completion [20]. These examinations allow for the systematic evaluation of acquired skills, including the operation of ultrasound machinery, fetal positioning, and the execution of measurements for gestational age determination [14, 32]. A pass in the practical examination necessitates the successful completion of a set number of tasks, with participants’ performances and the accuracy of their measurements benchmarked against those of seasoned professionals [14, 25].

Additional methods to assess the training’s impact include the review and discussion of images captured during practical sessions, formal assessments within the course framework by instructors, and the gathering of qualitative data through in-depth interviews and focus group discussions. These approaches provide nuanced insights into the participant’s experiences, attitudes, and any encountered challenges [14, 25, 27, 29].

For a comprehensive assessment of the sustainability of the skills and knowledge acquired, competency evaluations are recommended at intervals post-training, typically ranging from three to 6 months, and in some instances, extending up to a year. This longitudinal approach aids in determining the retention and application of the ultrasound competencies over time [14, 16, 24].

### What are potential challenges and problems in existing courses, and how can they be overcome? Are there any particular needs and expectations for the future development of obstetric ultrasound education?

The literature on the implementation of obstetric ultrasound training for midwives highlights several challenges and obstacles in the delivery process and participant training [17, 20]. A predominant focus is on short-term courses, which, despite their prevalence, are often criticized for insufficient duration that inadequately addresses the teaching of practical skills [16, 20, 22, 24]. This has led scholars like Bentley et al. and Zimmermann et al. to advocate for the exploration of more frequent and iterative educational interventions [16, 32]. Both the duration of courses and more opportunities for practical experience are considerations to be acknowledged [28].

A notable impediment is the inability of midwives to disengage from their professional duties during training, which, coupled with trainers’ concurrent clinical responsibilities, results in limited availability for essential supervised scanning sessions [20, 25]. Proposals have been made to negotiate dedicated time within work schedules to accommodate training needs [17].

Diversity in trainees’ pre-existing knowledge and experience presents another challenge, indicating a need for early and more frequent practical assessments to identify varying levels of aptitude and to provide tailored support [17, 28]. Solutions include segregating trainees into subgroups based on their proficiency levels and offering courses in native languages or with interpreter support to bridge language barriers [17, 20].

Longitudinal follow-up post-training is frequently lacking, limiting insights into the long-term impacts on patient care and clinical management [16, 24]. Future research is encouraged to address the effects of midwife-conducted ultrasound exams on patient outcomes [16]. In addition, uncertainties related to accreditation, eligibility, and guidelines for ultrasound use by non-radiologists highlight a broader issue of policy deficiencies that need addressing to enhance ultrasound accessibility and utilization [23].

In LMICs, where most obstetric ultrasound training research for midwives is conducted, progress is significantly hindered by factors like inadequate infrastructure, geographic isolation, and resource limitations. The absence of sustained supervision and mentorship post-training exacerbates these challenges, affecting the development and confidence in POCUS skills [17, 20]. Teleultrasound and formal accreditation in POCUS with longitudinal mentorship have been proposed as solutions to these challenges [20].

Moreover, high costs, equipment failures, and the poor quality of mobile ultrasound units, combined with limited local technical support, call for more affordable device options, thorough pre-distribution equipment inspections, and contingency plans for equipment malfunction [17, 20]. Infrastructure issues, such as unstable power supplies and limited internet access, further complicate the implementation of tele-ultrasound, underscoring the necessity of stable infrastructure to support the growing importance of ultrasound in LMICs [27, 33].

### Which applications or tools are available to simplify the education of obstetric ultrasound to midwives?

To enhance the proficiency of midwives in the application of obstetric ultrasound and facilitate the acquisition of relevant skills, several studies have explored the utility of simulators and specialized ultrasound systems. Ultrasound simulators have been recognized as effective educational tools, offering an alternative to clinical training environments, which are often constrained by time limitations and the practical challenges of working with patients [34–37]. Simulator-based learning (SBL) provides a controlled, stress-free environment that enables learners to practise extensively and engage in inquiry without the apprehensions associated with patient interactions. This approach also circumvents issues related to patient recruitment, discomfort during prolonged examinations, and the psychological impact of being observed by patients [21, 36].

Comparative studies assessing the efficacy of SBL have divided trainees into groups, with one having access to simulators and the other not. Findings indicate that simulators are beneficial for teaching fundamental ultrasound skills, offering a controlled learning environment that facilitates content delivery and enhances the rapidity and accuracy of ultrasound image acquisition [21, 34, 36, 37]. However, despite these advantages, final assessment outcomes do not demonstrate significant superiority over traditional methods, especially regarding long-term skill retention. Thus, integrating SBL with clinical practise is recommended to maximize competency development [21, 34].

Despite its benefits, SBL is not without drawbacks, including high operational costs and the inability to replicate certain real-life conditions such as amniotic fluid dynamics, fetal movements, and the manipulation of fetal position [21, 36]. Differences in the appearance of anatomical structures on simulators compared to actual ultrasound machines have also been noted, underscoring the necessity of complementing SBL with hands-on clinical experience to achieve comprehensive ultrasound proficiency [37].

Technological advancements have also been explored to support midwives’ training in obstetric ultrasound. Studies on the use of three-dimensional (3D) ultrasound technologies have aimed to facilitate the visualization of target anatomical structures, although the benefits appear to be limited to slight improvements in femoral length visualization at the expense of increased procedure time [38, 39].

Telemedicine has emerged as a promising tool to bridge geographical gaps, particularly between novice midwives and experts in maternal–fetal health. Implementations such as web-based telemedicine systems, incorporating diagnostic devices and digital communication tools, have shown potential in enhancing the transmission of clinical data and images, as well as providing real-time guidance on ultrasound image acquisition and interpretation [35].

Integrated learning tools and tele-radiology systems have been developed to support midwives further, offering theoretical resources, decision-making aids, and the capability to transmit images for expert review [18, 27, 39]. Despite high levels of satisfaction with these technological supports, challenges related to electrical and internet reliability may impede widespread adoption [27].

## Discussion

This manuscript describes the significance and operationalization of obstetric ultrasound education for midwives, underlining its potential to substantially elevate the quality of maternal care. Existing literature and the current paradigm of ultrasound education in midwifery education were discussed. Several key elements being essential for an effective integration and sustainability of ultrasound training programs in midwifery education are presented.

The incorporation of obstetric ultrasound into midwifery practise empowers midwives to deliver a more holistic care model to pregnant women [15]. This is particularly salient in remote and LMICs, where enhancing prenatal care resources through point-of-care ultrasound (POCUS) and telemedicine can significantly mitigate prevalent healthcare challenges, including high maternal and fetal mortality rates [17, 20, 30]. POCUS, characterized by its portability and independence from traditional clinical settings, can alleviate economic burdens associated with accessing ultrasound facilities [17].

Educational programs in obstetric ultrasound typically encompass theoretical and practical dimensions, with an emphasis on hands-on training. This practical component, often facilitated through engagement with pregnant volunteers, is critical for transitioning to clinical phases where midwives apply their skills under supervision [17]. In addition, the role of simulator-based learning (SBL) as a controlled and efficacious educational strategy is increasingly acknowledged within formal training contexts [37].

Despite these advancements, the initiation of ultrasound training within midwifery curricula encounters several challenges. A recurrent critique pertains to the insufficient duration of courses, which compromises the depth of practical skill development. The varied expertise amongst participants further complicates course delivery [22, 28]. Moreover, the absence of longitudinal follow-up post-training limits insights into the enduring impacts on patient care and maternal and child health outcomes [16]. The lack of standardized curricula, clear guidelines, and political backing further constrains the efficacy of ultrasound education [23, 24]. Specifically, in LMICs, infrastructural, technical, and personnel deficiencies impede the realization of educational goals [20, 27].

Hence published recommendations emerge for optimizing obstetric ultrasound courses for midwives, by extending course durations to adequately cover essential content, reducing group sizes to enhance hands-on learning opportunities, and incorporating more frequent educational sessions [16, 22, 28]. Longitudinal participant observation should be conducted to elucidate the impact of midwife-conducted ultrasound exams on healthcare outcomes [16, 24]. Adapting lectures to different levels of pre-existing knowledge and employing early and regular OSCEs can further improve the efficiency and effectiveness of learning [17, 20, 28]. The integration of SBL with clinical practise is advocated to achieve comprehensive ultrasound competency and also alternative instructional modalities, such as online teaching, offer flexible learning opportunities, especially in remote areas [20, 30, 33, 37]. Furthermore, enhancing logistical support for accessing ultrasound training facilities, as well as ensuring dedicated time for midwives to engage in educational activities without workplace constraints can extend educational accessibility [20, 30].

Design of ultrasound training programs for midwives seems to be a key factor for improving the quality of midwifery care. However, its proof concept and feasibility in clinical practise must first be evaluated.

While some midwives see benefits in the use of ultrasound, others suggest significant burdens. Considering the use of ultrasound to be a potential factor for medicalization, pregnancies might be at “risk” for additional or even unnecessary interventions [43, 44]. Such concerns need to be acknowledged.

Our systematic literature review shows that most findings emerge from initiatives in LMICs outside Europe (Fig. 3). Focusing on local challenges to enhance prenatal care quality, conditions of midwives are markedly different from industrialized nations like Germany. As the range of tasks characterizes the scope of training, implementation of programs are in accordance with local regulations. Benefits and improvements of perinatal care cannot easily be transferred from developing country to industrialized, or vice versa.

Focusing on industrialized countries, large distances between rural regions and specialized birth centers, with high-risk pregnancies might by supported. Thus enhance the access to essential prenatal diagnostics for underserved areas and contributing to the improvement of prenatal care. Moreover, considering the frequently high workload in clinical settings, promoting interdisciplinary collaboration between midwives and obstetricians/gynecologists could be facilitated by midwives assuming sonographic responsibilities [45]. This capability becomes particularly valuable in obstetric emergency scenarios, where midwives could provide prompt and efficient initial evaluations. Such a shift not only improves the quality of the midwife’s prescribed activities but also integrates seamlessly with the ongoing academization of the profession in Germany. Reflecting this trend, since 2020, numerous German cities have seen the establishment of bachelor’s degree programs in midwifery as part of the academization process [46]. Consequently, incorporating ultrasound training for midwives in Germany would happen within or alongside these bachelor’s degree programs. However, a close look at the curriculum of the degree program reveals that the predetermined schedule limits in-depth experience with prenatal care, particularly for high-risk pregnancies. Thus, it’s essential to consider alternative approaches to optimize the instruction of ultrasound training within Germany’s midwifery education system. Incorporating ultrasound training into a Master’s degree course could be an effective strategy to prevent adding to the academic burden within current study structures and to ensure ultrasound training is not merely a voluntary option. This approach could offer further training as an Advanced Practical Midwife, allowing for a deeper and more focused engagement, more time to gain experience and enhancing quality assurance. Simultaneously, a standardized curriculum is necessary to streamline the educational process and to ensure uniform knowledge of ultrasound techniques and their application in maternal and fetal health. Collaborative effort is essential amongst educational institutions, professional associations, healthcare policymakers, regulatory bodies, and practitioners including midwives, obstetricians, and ultrasound experts. In this context of creating a cohesive educational framework, it is vital to proactively assess anticipated expenses, including equipment costs and their integration into daily practise, as well as to explore options for financial coverage. Without specific cost data, discussions remain speculative and could lead to resistance, especially amongst freelance midwives. Current maternity care guidelines do not cover funding for ultrasound examinations by midwives, creating a policy gap that may limit the use of this diagnostic tool.

In case of responsibility toward pregnant woman, particularly with regard to alternative approaches such as care via telemedicine, it is imperative to establish clear, binding guidelines that define the scope, roles, and responsibilities of each profession involved, ensuring a harmonious and efficient collaboration. Especially role allocation in ultrasound between midwives and obstetricians/gynecologists (OB-GYNs) remains a central topic, which will subsequently influence the midwifery curriculum. In this context, it is important to emphasize that ultrasound training for midwives cannot be compared to the extensive and detailed training that OB-GYNs receive, particularly regarding quality. The purpose of introducing ultrasound to midwives is not to supplant or replace the medical profession but, as already mentioned, to improve the quality of midwifery work. However, this particular matter—the uncertain future trajectory of ultrasound training for obstetrics/gynecology, as recently explored in the publication by Recker et al. [45]—remains a topic of intense debate and significant interest. Nevertheless, the evolution of midwifery education and profession is present and it is foreseeable that midwives will increasingly provide antenatal care. Therefore, it is crucial to examine the benefits of ultrasound training in adapting to these changes and future direction of midwifery to equip midwives for the evolving demands of prenatal care [45].

Further research and dialogue are needed to assess the opportunities, as well as the efficacy and sustainability of ultrasound training within midwifery education in Germany, focusing on standardized curriculum development and addressing financial and regulatory challenges. It is crucial to determine the long-term impacts of ultrasound training on the midwifery profession and its evolving role in prenatal care. In addition, a clear delineation of roles and responsibilities between midwives and other healthcare professionals is necessary to ensure that ultrasound is used effectively and appropriately in maternal care, optimizing outcomes for pregnant women.

This systematic review acknowledges its limitations, given the dynamic nature of research in obstetric ultrasound education for midwives, and recognizes the potential for new studies to have emerged during its compilation.

## Conclusion

The provision of obstetric ultrasound training to midwives represents a pivotal advancement in prenatal care, significantly bolstering their ability to deliver comprehensive care to both mothers and newborns. In the quest for optimizing educational outcomes, a diverse array of pedagogical strategies and learning modalities has been explored. Enhancements in the structure of such training, specifically through the extension of course durations and increased frequency of sessions, alongside sufficient allotment for hands-on practise, are posited to substantially improve midwives’ ultrasound proficiency. Moreover, the implementation of tailored group formations, based on competency levels, can further refine learning efficiency.

Critical to the transition of trainees into clinical settings is the provision of robust mentorship and supervisory frameworks, ensuring the practical application of acquired skills under guided oversight. Future research endeavors should pivot towards evaluating the long-term ramifications of ultrasound training on clinical outcomes, thereby substantiating its impact on maternal and neonatal health.

Nevertheless, the landscape of ultrasound education for midwives is marred by systemic challenges, necessitating policy reforms and the adoption of standardized training curricula to navigate through these complexities effectively. These challenges extend beyond educational frameworks, encompassing financial difficulties and the broader implications of integrating ultrasound training into the midwifery profession and its pivotal role in prenatal care. Addressing these existing barriers is imperative for creating an educational ecosystem that is effective, consistent, and sustainable. As we continuously refine and expand ultrasound training paradigms for midwives, it becomes evident that such efforts mark a significant stride towards improving the quality of maternal and neonatal healthcare services.

## Data Availability

Data available on request from the authors.
